# Alpha thalassaemia-mental retardation, X linked

**DOI:** 10.1186/1750-1172-1-15

**Published:** 2006-05-04

**Authors:** Richard Gibbons

**Affiliations:** 1 MRC Molecular Haematology Unit, Weatherall Institute of Molecular Medicine, John Radcliffe Hospital, University of Oxford, Headington, OX3 9DS Oxford, UK

## Abstract

X-linked alpha thalassaemia mental retardation (ATR-X) syndrome in males is associated with profound developmental delay, facial dysmorphism, genital abnormalities and alpha thalassaemia. Female carriers are usually physically and intellectually normal. So far, 168 patients have been reported. Language is usually very limited. Seizures occur in about one third of the cases. While many patients are affectionate with their caregivers, some exhibit autistic-like behaviour. Patients present with facial hypotonia and a characteristic mouth. Genital abnormalities are observed in 80% of children and range from undescended testes to ambiguous genitalia. Alpha-thalassaemia is not always present. This syndrome is X-linked recessive and results from mutations in the *ATRX *gene. This gene encodes the widely expressed ATRX protein. *ATRX *mutations cause diverse changes in the pattern of DNA methylation at heterochromatic loci but it is not yet known whether this is responsible for the clinical phenotype. The diagnosis can be established by detection of alpha thalassaemia, identification of *ATRX *gene mutations, ATRX protein studies and X-inactivation studies. Genetic counselling can be offered to families. Management is multidisciplinary: young children must be carefully monitored for gastro-oesophageal reflux as it may cause death. A number of individuals with ATR-X are fit and well in their 30s and 40s.

## Disease name and synonyms

X-linked alpha thalassaemia mental retardation (ATR-X) syndrome.

## Definition/diagnostic criteria

Since the identification of the causative gene (*ATRX*) and the advent of procedures to confirm the diagnosis by molecular means, it has become clear that there are few *sine qua non *diagnostic features. Generally, however, affected individuals have moderate to profound learning difficulties associated with a severe expressive language disorder (95% of cases have severe to profound mental retardation (MR)). Most cases (>90%) will have a characteristic and recognisable facial gestalt during infancy. Some degree of genital abnormality is common (80%). Many affected individuals (90%) will have haematological signs of alpha thalassaemia. Other features include: skeletal abnormalities (90%), microcephaly (75%), short stature (65%), seizures (30%), cardiac defects (20%), renal/urinary abnormalities (15%).

## Epidemiology

The prevalence of ATR-X in the general population is unknown. So far, 168 patients have been reported. An estimate for the prevalence is <1-9/1,000,000.

## Clinical description

### Psychomotor retardation and central nervous system

Most cases of ATR-X syndrome have global developmental delay. However, even in less severely affected cases, where general performance may be classified as in the moderate level, expressive language is usually very limited.

In early childhood generalised hypotonia is common and all milestones are delayed. More severely affected patients do not walk until later in childhood and some never ambulate. Most have no speech, although an increasing number of individuals are being identified who use a few words or signs. The most severe cases may only have situational understanding, and most are dependent on others for almost all daily activities. More recent reports, however, point to a wider spectrum of intellectual handicap than previously thought. A mutation in the *ATRX *gene has recently been identified in family originally described by Carpenter and colleagues [1]. All affected males had moderate MR and exhibited expressive language delay, though no psychometric evaluation was available. Guerrini and colleagues reported a mutation in an Italian family with four affected male cousins, one had profound MR, whereas the others had an intellectual quotient (IQ) of 41, 56 and 58 [2]. The basis for this marked variation is unknown.

Generally, affected individuals continue to acquire new skills, though a brief period of neurological deterioration has been reported in three cases. In one report, electroencephalogram (EEG) changes were consistent with encephalitis [3]. The family originally reported by Holmes and Gang [4] was subsequently shown to have an *ATRX *mutation [5]. The reported family consisted of an infant and two maternal uncles with microcephaly, large fontanel, hypotonic face with short nose and anteverted nares, epicanthus, club foot deformity, and retarded psychomotor development. All three affected males from this family died in childhood and the death of one was attributed to encephalitis.

With age, affected individuals often develop a tendency toward spasticity. One report described a family with an *ATRX *mutation where affected members had spastic paraplegia from birth [6].

Seizures occur in approximately one third of cases and most frequently are clonic/tonic or myoclonic in nature. A number of parents have reported jerking movements, which are not associated with epileptiform activity on EEG.

Assessment of vision and hearing is difficult. Vision usually appears normal, although two patients have been reported as blind. Optic atrophy or pale discs are commonly noted, as are refractive errors (especially myopia). Sensorineural deafness and alpha thalassaemia were previously considered as features that distinguishes ATR-X syndrome from the allelic condition Juberg-Marsidi syndrome [7]. However, of the 13 cases with a documented sensorineural hearing deficit, seven had alpha thalassaemia suggesting the two conditions are part of a single continuum.

Although the head circumference is usually normal at birth, post-natal microcephaly usually develops. Macrocephaly has not been reported.

Computed tomography (CT) and magnetic resonance imaging (MRI) do not generally reveal remarkable findings, although mild cerebral atrophy may be seen. In two cases, partial or complete agenesis of the corpus callosum was reported. Autopsy reports were available for only three cases. The brain was small in each; in two cases the morphology was normal, but in the remaining case the right temporal gyri were indistinct and there was hypoplasia of the cerebral white matter.

### Behavioural phenotype

No systematic study of behaviour has been carried out for ATR-X syndrome. Consequently, most reports of behavioural characteristics are anecdotal. Nevertheless, a thumbnail sketch of the mannerisms of this condition is slowly emerging [8,9] and may be useful for diagnosis.

The subjects are usually described by their parents as content and of a happy disposition. Affected individuals exhibit a wide range of emotions that are usually appropriate to their circumstances. There have been reports, however, of unprovoked emotional outbursts with sustained laughing or crying. There may be emotional fluctuation with sudden switches between almost manic-like excitement or agitation, and withdrawal and depression. In several instances, the episodes of crying have been thought to be associated with pain, possibly of a gastrointestinal origin (see below).

Whereas many of the individuals are affectionate to their caregivers and appreciate physical contact, some patients exhibit autistic-like behaviour: patients appear to be in a world of their own, show little interest or even recognition of those around them, and avoid eye contact. The latter behaviour may be associated with an unusual and persistent posture.

Affected individuals may be restless, exhibiting choreoathetotic-like movements. Frequently, they put their hands into their mouths and may induce vomiting. Sometimes, they engage in self-injurious behaviour, biting or hitting themselves. They may hit, push or squeeze their neck with their hands to the point of cyanosis, a state they may also achieve through breath-holding. They may exhibit obsessional behaviour such as spinning on one spot while gazing into a light. Repetitive stereotypic movements may be manifest and these may vary from pill-rolling to hand flapping. This characteristic behaviour is reminiscent of Angelman syndrome and may lead to diagnostic confusion.

### Facial anomalies

**Figure 1:**
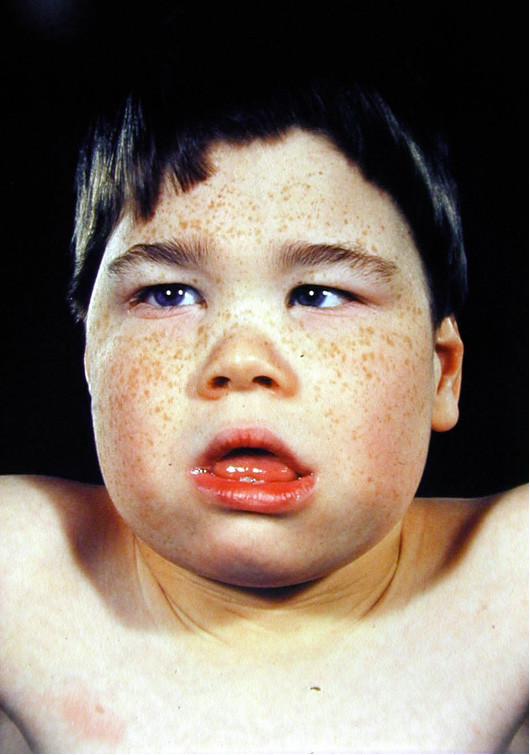
Child of 8 yrs with the characteristic facial features of ATR-X syndrome. Note the upswept frontal hair line, hypertelorism, epicanthic folds, flat nasal bridge, small triangular upturned nose, tented upper lip, everted lower lip and hypotonic facies.

Distinctive facial traits are most readily recognised in early childhood and the gestalt is probably secondary to facial hypotonia (Figure 1). The frontal hair is often upswept, there is telecanthus, epicanthic folds, flat nasal bridge and mid-face hypoplasia, and a small triangular upturned nose with the alae nasi extending below the columella and septum. The upper lip is tented, the lower lip full and everted. The frontal incisors are frequently widely spaced, the tongue protrudes and there is prodigious dribbling. The ears may be simple, slightly low set and posteriorly rotated.

### Genital abnormalities

Genital abnormalities are seen in 80% of children. These may be very mild (*e.g*., undescended testes or deficient prepuce), but the spectrum of abnormality extends through hypospadias and micropenis to ambiguous female external genitalia. The most severely affected children, who are clinically defined as male pseudohermaphrodites, are usually raised as females. In such cases there are no Mullerian structures present, and intra-abdominal, dysgenetic testes or streak gonads have been found [10,11]. Of particular interest is the finding that these abnormalities breed true within families [12]. Puberty is frequently delayed and, in a few cases, appears to be arrested. Curiously, premature adrenarche has been noted in two children ([13] and unpublished observation).

### Skeletal abnormalities

In a series of 45 cases, a wide range of relatively mild skeletal abnormalities were noted, some of which were probably secondary to hypotonia and immobility [14]. Fixed flexion deformities, particularly of the fingers, were common. Other abnormalities of the fingers and toes were also observed: clinodactyly, brachydactyly, tapering of the fingers, drum stick phalanges, cutaneous syndactyly, overlapping of the digits, and a single case with a bifid thumb. Foot deformities occured in 29% of cases and include pes planus, talipes equinovarus and talipes calcaneovalgus. Almost a third of the cases had kyphosis and/or scoliosis, and chest wall deformity was seen in 10 cases. Sacral dimples were present in three cases, radiological spina bifida in two cases and other abnormalities of the vertebrae in five cases. Only a few of the cases had a thorough radiological investigation. In those investigated, the most common findings were delayed bone age and coxa valga. Short stature was seen in two-thirds of cases. Longitudinal data were available for only a few cases. As noted previously, in some patients growth retardation is apparent throughout life, whereas in others it manifests at a later stage, *e.g*. at the time of the pubertal growth spurt.

### Miscellaneous abnormalities

Recurrent vomiting, regurgitation or gastro-oesophageal reflux, particularly in early childhood, are common findings. In a recent study involving a pair of affected non-identical twins, a barium meal revealed that both children had episodic gastric pseudovolvulus. In this condition the stomach does not have the normal system of peritoneal ligaments and has a propensity to torsion around itself leading to gastric outlet obstruction and secondary gastro-oesophageal reflux [15]. An apparent reluctance to swallow has been reported by several parents and probably reflects the dyscoordinated swallowing that was observed radiologically in two well-studied cases (unpublished data). The tendency for aspiration is commonly implicated as a cause of death in early childhood. Excessive drooling is very common, as is frequent eructation. Constipation occurs often, and in some individuals is a major management problem. Martucciello *et al*. [15] demonstrated ultra-short Hirshsprung's disease and colonic hypoganglionosis in two affected children. The authors reviewed 128 cases of ATR-X and found that hospital admissions for recurrent ileus were reported in two cases and that reduced intestinal mobility was observed radiologically in four cases. This may be a consequence of a widespread abnormality in the enteric nervous system, leading to abnormal gut motility. Two of the patients required partial resection of the ileum after developing ischaemia of the small bowel, which in one case was attributed to a volvulus. Volvulus was also reported in an additional case. One child required a right hemicolectomy following an episode of necrotising enterocolitis at 13 days of age (reviewed in [15]).

Evidence suggests that affected individuals are susceptible to peptic ulceration. Oesophagitis, oesophageal stricture and peptic ulcer have observed endoscopically in single cases. In five cases, an upper gastrointestinal bleed was observed, one of which required transfusion (haemoglobin, 5 g/dl) [14,15]. Pain resulting from peptic ulceration is one possible explanation for the episodes of persistent crying and food refusal reported by a number of parents. A wide range of cardiac abnormalities have been noted: septal defects (10 cases); patent ductus arteriosus (six cases); pulmonary stenosis (three cases); aortic stenosis (two cases); tetralogy of Fallot (two cases) and single cases of transposition of the great arteries, dextracardia with situs solitus and aortic regurgitation ([14] and unpublished data).

Renal abnormalities (hydronephrosis, renal hypoplasia or agenesis, polycystic kidney, vesico-ureteric reflux) may present with recurrent urinary tract infections.

### Haematology

Although the presence of alpha thalassaemia was initially one of the defining elements of the syndrome, it is clear that there is considerable variation in the haematological manifestations associated with *ATRX *mutations. A number of families have been identified in which some or all of the affected members show no signs of alpha thalassaemia [16,17]. The test for alpha thalassaemia is simple and, when positive, quickly establishes the diagnosis (see diagnostic methods). However, the haematological findings are often surprisingly normal considering the presence of alpha thalassaemia. Neither haemoglobin concentration nor mean cell haemoglobin concentration are as severely affected as in the classical forms of alpha thalassaemia associated with cis-acting mutations in the alpha globin complex. This discrepancy probably reflects the different pathophysiology of the conditions.

## Aetiology

The gene involved in the disease, *ATRX*, lies at Xq13.3 [18]. It spans about 300 kb of genomic DNA and contains 36 exons [19]. It encodes at least two alternatively spliced ~10.5 kb mRNA transcripts which differ at their 5' ends and are predicted to give rise to slightly different proteins of 265 and 280 kD respectively. A further transcript of ~7 kb represents an isoform which retains intron 11 and truncates at this point. This gives rise to a truncated protein isoform, ATRXt, which is conserved between mouse and man [20]. The protein belongs to the SNF2 family of helicase/ATPases, members of which are involved in a wide variety of cellular functions including the regulation of transcription (SNF2, MOT1 and brahma), control of the cell cycle (NPS1), DNA repair (RAD16, RAD54 and ERCC6) and mitotic chromosome segregation (lodestar). It is believed that their function is to facilitate these processes by remodelling chromatin. Another important feature of the ATRX protein is the presence, at the N terminal, of a zinc finger domain (called the ADD domain), which is related to sequences seen in the DNA methyltransferase 3 (DNMT3) family of *de novo *DNA methyltransferases [21]. It is not known whether this domain is involved in protein or DNA interactions. The majority of naturally occurring mutations in the *ATRX *gene occur in the ADD and helicase domains [22]. It is likely that all the mutations are associated with reduced function.

The function of the ATRX protein is unknown, but the fact that alpha globin expression is perturbed in the patients suggests that it may play a role in gene expression. Protein studies have shown that ATRX is a nuclear protein with a punctate staining pattern [23]. In mouse cells, and to a lesser extent in human cells, the majority of the protein is associated with DAPI-bright regions of the nucleus, which are known to represent pericentromeric heterochromatin. Evidence is also accumulating for an interaction between ATRX and another heterochromatic protein, HP1 [24,25]. ATRX is also found in promyelocytic leukaemia nuclear bodies, where it interacts with the transcription cofactor Daxx [26,27]. One additional striking finding in human metaphase preparations is that anti-ATRX antibodies consistently localise to the short arms of acrocentric chromosomes and co-localise with a transcription factor (upstream binding factor) that is known to bind the ribosomal DNA (rDNA) arrays in nucleolar organiser regions [23].

The effects of *ATRX *mutations on the chromatin structure of the rDNA arrays located in these regions have been studied. Although no gross changes in DNAase1, micrococcal nuclease or endonuclease accessibility were detected, striking differences were noted in the pattern of rDNA methylation between normal controls and patients with ATR-X syndrome [28]. In normal individuals, approximately 20% of the transcribed units were methylated; whereas, in ATR-X patients, a substantial number of these rDNA genes were unmethylated. An extensive survey of the genome has identified two additional sequences that are abnormally methylated in ATR-X patients. Y-specific repeats (DYZ2) are almost all methylated in ATR-X patients, while ~6% are unmethylated in peripheral blood of normal individuals. Subtle changes in the pattern of methylation have also been observed in the TelBam3.4 family of repeats that are mainly found in the subtelomeric regions. It is not clear whether the perturbation of methylation that is observed in affected individuals plays a role in the aetiology of the condition. To date, no change in the pattern of methylation has been detected in the alpha globin gene cluster that might explain the reduced expression of the alpha globin genes compared with expression of the beta globin genes.

In the knock-out mouse model, absence of ATRX is lethal early in embryogenesis indicating that ATRX is essential for development [29]. In conditional knock-out mice, where only the forebrain is affected, there was normal proliferation of cortical progenitors but widespread apoptosis associated with the differentiation of the cortical neurones, which led to a reduction in forebrain size [30].

## Genotype/phenotype correlations

Since the discovery of the *ATRX *gene, most new cases have been defined on the basis of severe MR with the typical facial appearance (see clinical description) associated with a mutation in the *ATRX *gene. This allows a less biased evaluation of the effect of *ATRX *mutations on the commonly associated clinical manifestations. The severity of three aspects of the phenotype, mental retardation, genital abnormality and alpha thalassaemia, is quantifiable to some degree.

The greatest variation in intellectual handicap is associated with a truncating mutation at the N-terminus of the protein [2]. The same mutation has also been described in the Chudley-Lowry family [31] in which the phenotype was milder than that usually associated with *ATRX *mutations. Protein analysis by Western blotting has shown that small amounts of full-length protein are present in each patient [31,32]. There is some debate about how this phenotypic rescue is achieved: the possibilities being skipping of the mutation by initiation at a downstream methionine or by alternative splicing. There is, however, no obvious correlation between the degree of retardation and the amount of full-length protein.

There are now eight different mutations associated with the most severe urogenital abnormalities. Five of these mutations lead to the production of a truncated protein resulting in the loss of the C-terminal domain, including a conserved element and polyglutamine tract. From the available data, it appears that in the absence of the C-terminal domain, severe urogenital abnormalities are likely (though not inevitable as one mutation in this region was associated only with cryptorchidism) suggesting that this region may play a specific role in urogenital development. At other mutation sites, however, there is no obvious link between phenotype and genotype and there is considerable variation in the degree of abnormality seen in individuals with identical mutations.

The relationship between *ATRX *mutations and alpha thalassaemia is unclear. Past reports are inevitably biased as the presence of excess beta chains (HbH inclusions) was originally used to define the ATR-X syndrome. However, inclusions may not appear until there is a 30–40% reduction in alpha chain synthesis [33]. Thus, some patients do not have HbH inclusions [16,17,34], but this does not rule out down-regulation of alpha globin expression. Furthermore, comparison of the 32 cases from 26 pedigrees with the common 736C>T mutation shows a variation in the frequency of HbH inclusions from 0 to 14%. This variable frequency of cells with HbH inclusions indicates considerable variability in the degree to which alpha globin synthesis is affected by the 736C>T mutations. The fact that patients with identical mutations may have very different, albeit stable, degrees of alpha thalassaemia suggests that the effect of the ATRX protein on alpha globin expression may be modified by other genetic factors.

## Diagnostic methods

### Detection of alpha thalassaemia

**Figure 2:**
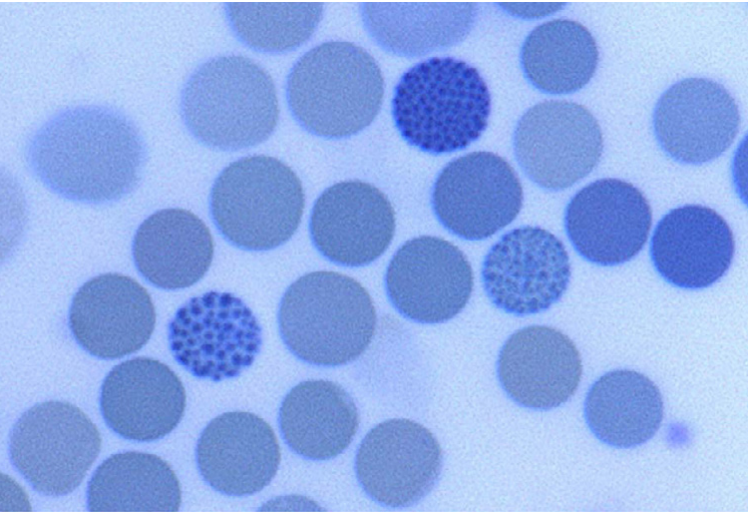
Slide of red blood cells after incubation in brilliant cresyl blue. Three cells have Haemoglobin H inclusions giving the cells a golf ball like appearance.

The most sensitive test uses light microscopy to detect red cells containing HbH inclusions after incubation of venous blood with 1% brilliant cresyl blue in isotonic saline for 4–24 hours at room temperature (Figure 2). HbH is unstable and cells with inclusions may be more difficult to find in older blood samples. When the family history and phenotype are highly indicative of ATR-X syndrome, a careful search for inclusions should be performed in all affected individuals and repeated, if necessary, as inclusions may be very infrequent. It is important to note that, in most cases of ATR-X, the amounts of HbH are too low to be detected by electrophoresis. The haematology is often surprisingly normal considering the presence of alpha thalassaemia.

### Mutation detection

The majority of disease-causing mutations are single base changes, most of which are missense mutations. The gene is large and a comprehensive mutation analysis, even of the coding region alone, is a time-consuming and expensive enterprise. However, the missense mutations are clustered: 50%–60% of the identified mutations are located in the ADD domain, 20% have the common 736C>T, R246C mutation and the next most commonly affected region is the helicase domain. A sensible and cost-saving approach is to start by analysing these regions.

### Protein studies

Levels of ATRX protein were found to be substantially reduced in a number of patients with missense mutations involving the ADD domain [23,35]. Analysis of the ATRX protein may also reveal a truncation in the ATRX protein of affected individuals, which allows more directed sequencing.

### X inactivation studies

X-chromosome inactivation studies may be used to determine the carrier status of at-risk women. In normal women, one X-chromosome is randomly inactivated in each cell. Most females who are unaffected carriers for ATR-X syndrome display preferential inactivation of the mutant *ATRX *allele [36]. This phenomenon is referred to as "non-random" or "skewed" X-chromosome inactivation and can be identified by established laboratory techniques. There are, however, exceptions: a child of normal intelligence carrying an *ATRX *mutation was shown to have a balanced pattern of X inactivation [36]; another female carrier with mild mental retardation also had a non-skewed pattern of X inactivation [37]. This indicates that there may be a risk of false negative results associated with this test. Furthermore, 10% of normal females show skewed X-chromosome inactivation, which could lead to false positives. Finally, in women with certain X-linked conditions, other than ATR-X syndrome, the X chromosome carrying the mutated gene is preferentially inactivated [38]. Since non-random X-chromosome inactivation is not unique to ATR-X syndrome, the test is only supportive, to be used in the context of a suggestive clinical and/or family history.

## Differential diagnosis

Coffin-Lowry syndrome may be confused with ATR-X syndrome, particularly in early childhood. Distinguishing features are the down-slanting palpebral fissures, broad nose, pudgy tapering digits, absence of genital abnormalities and the frequent presence of carrier manifestations in Coffin-Lowry syndrome. There is phenotypic overlap with Angelman syndrome (profound MR with absent speech and walking, seizures, happy disposition, emotional lability) and Smith-Lemli-Opitz (facial dysmorphism, skeletal and genital abnormalities). Diagnostic testing or mutational analysis should allow these conditions to be excluded in most cases.

Several other syndromes, characterised by the association of severe mental retardation with dysmorphic features, are caused by allelic mutations in the *ATRX *gene: Juberg-Marsidi [34], X-linked mental retardation with spastic paraplegia [6], Carpenter-Waziri [39], Holmes-Gang [5], Smith-Fineman-Myers [40] and Chudley-Lowry [31] syndromes.

## Genetic counselling and antenatal diagnosis

ATR-X syndrome is a recessive X-linked condition. Female carriers are usually physically and intellectually normal and therefore additional tests are required to determine a female's genotype. In the past, skewed X-inactivation has been utilised as a marker for carriers of the *ATRX *mutation; however, for the reasons given above, this method should be used with caution. Twenty-five percent of obligate female carriers exhibit rare cells with HbH inclusions, and thus a negative result does not exclude carriage of a mutation. Since the identification of the *ATRX *gene, mutation detection has become the mainstay of carrier identification. In families in which the causative mutation has not been identified, linked markers may be used to determine whether descendants of an obligate carrier have inherited the disease-associated haplotype.

For a woman identified as a carrier, there is a 50% risk of passing on the disease allele but, since only males are clinically affected, the risk of having an affected child is 25% for each pregnancy. Antenatal diagnosis for such at-risk females is feasible.

The principal issue when counselling is determining the risk of recurrence for families with a sporadic case of ATR-X. One small study showed that 17/20 mothers of sporadic cases were carriers [41]. Therefore, in families where the mutation has not been identified, 85% of the mothers of sporadic cases would be expected to be carriers.

Another important consideration concerns the possibility of germline mosaicism. This has been recently reported in ATR-X syndrome and means that, despite a negative mutation test, a mother of an ATR-X patient may still be at risk of further affected offspring [41]. It is advisable to offer all mothers of affected children prenatal diagnosis even if they are negative for the *ATRX *mutation.

## Management including treatment

### Development and behaviour

Evaluation of developmental skills from infancy ensures that the appropriate intervention services are introduced as early as possible. Infant stimulation, early intervention and special education are important to optimise abilities. Affected individuals may show improvement in socialisation with one-to-one therapy. Anti-psychotic medication such as prochlorperazine may be effective in treating severe behaviour problems.

### Feeding and gastro-intestinal problems

Infantile hypotonia is very common and is associated with considerable difficulty with sucking. Gavage feeding may be required for a number of weeks to assure adequate nutrition.

Recurrent vomiting, regurgitation or gastro-oesophageal reflux, particularly in early childhood, is a common finding and may be secondary to gastric pseudovolvulus. Evaluation of recurrent vomiting should include assessment for gastro-oesophageal reflux and there is a strong case for 24 hr pH monitoring and barium study of the upper gastro-intestinal tract. Initial treatment for gastro-oesophageal reflux should be conservative and standard. Nutrition consultation for assurance of adequate caloric intake may be needed. In severe cases, surgical treatment by fundoplication may be indicated, as well as the introduction of a feeding gastrostomy. If gastric pseudovolvulus is demonstrated this requires surgical correction.

Prolonged periods of food and drink refusal associated with considerable distress are frequently reported by parents. The cause is rarely obvious, but peptic ulceration, oesophagitis or ileus should be considered, as these are well documented in this condition.

Constipation can be a significant management problem and fecal impaction may occur if preventative measures are not undertaken. Adequate hydration is important in preventing constipation, as is the use of bulking agents in the diet and the regular use of osmotic laxatives such as lactulose. If the problem is resistant to conservative management then rectal biopsy should be considered to exclude ultra-short segment Hirshsprung's disease and colonic hypoganglionosis.

Drooling is very common in ATR-X, particularly in young children. Many mothers will describe their sons soaking several bibs during the course of the day. The open mouth associated with facial hypotonia no doubt is an important factor, as is their reluctance to swallow even with a mouth full of saliva. Numerous methods have been tried to control drooling. Feeding by mouth may help encourage the swallowing reflex and this may be supplemented by specialist training by a speech therapist. In other conditions in which there is drooling, anticholinergic drugs are commonly used to reduce production of saliva. However, these drugs cause reduced gastrointestinal motility (which may already be abnormal in ATR-X males) and may exacerbate constipation or provoke ileus. Botulinum toxin type A (Botox) injection of the salivary glands might be tried, but reports are scant and the treatment needs repeating. The surgical options of redirecting the submandibular ducts or removing the glands themselves may be considered.

### Neurologic

Spasticity may increase with age. Regular evaluations of the need for physical therapy are needed and ongoing therapy may ameliorate spasticity.

Seizures occur in approximately one third of cases and most frequently are clonic/tonic or myoclonic in nature. In the majority of cases, seizures respond well to standard therapy. Some affected individuals exhibit jerking movements which, though appearing to be seizures, are not associated with epileptiform activity on EEG. Electroencephalogram may need to be carried out with video recording to correlate seizure activity and abnormal movements. Approach to seizure control is standard.

### Haematology

In ATR-X syndrome about 90% of cases have alpha thalassaemia. The anaemia, however, is mild and does not require treatment. Iron is not indicated unless iron stores are shown to be low.

### Genitourinary

Genital abnormalities are seen in 80% children. The possibility of cryptorchidism should be assessed in all affected children. Orchidopexy should be carried out as required at the standard age. Intra-abdominal testes, which are usually dysgenetic, should be removed because of the long-term risk of malignancy.

Structural abnormalities of the kidneys and ureters are well described and may predispose individuals to urinary tract infections. Renal abnormalities (hydronephrosis, renal hypoplasia or agenesis, polycystic kidney, vesico-ureteric reflux) may present with recurrent urinary tract infections. The urinary system should be imaged with ultrasound at diagnosis. Urine should be cultured when there is symptomatology such as fever or pain on urination. Prophylactic antibiotics are indicated if urinary tract anomalies are present to prevent long-term damage to the kidneys.

### Musculoskeletal

A wide range of relatively mild skeletal abnormalities have been noted, some of which are probably secondary to hypotonia and immobility [14]. Foot deformities occur in 29% of cases and include pes planus, talipes equinovarus and talipes calcaneovalgus. Kyphosis and scoliosis are common and increase with age. A careful musculoskeletal exam should be done at diagnosis and throughout childhood. Treatment of musculoskeletal anomalies, when appropriate, is standard.

### Hearing and vision

Sensorineural deafness may be present. Standard distraction tests and, if anomalies are suspected, evaluation of auditory evoked responses should be done. Hearing loss should be managed as for any infant.

Refractive abnormalities, in particular myopia, are common. Strabismus may be present. Rarely the children may be blind and this may be associated with optic atrophy. A formal ophthalmologic evaluation is appropriate at diagnosis and regularly thereafter. Ocular problems should be treated as in the general population.

## Prognosis

Among the 168 patients described, there have been 25 deaths. The cause was established in just over half of these cases: there were six cases of pneumonia and four due to aspiration of vomitus. Patients appear to be particularly vulnerable in early childhood, with 19 of the deaths occurring under the age of five years. This may be associated with the fact that gastro-oesophageal reflux and vomiting are often more severe in the early years. In two cases, death was associated with renal failure, which was possibly due to recurrent renal tract infection. There are no long-term longitudinal data for this relatively newly described syndrome but a number of affected individuals are fit and well in their 30s and 40s.

## Unresolved questions

1. The function of ATRX protein is not known. It is not clear how loss of function mutations in a protein associated with repressive heterochromatin might lead to reduced expression of a target gene (*e.g*. alpha globin). The role of ATRX in DNA methylation is also unknown.

2. What are the 'target' genes whose expression is directly influenced by ATRX? How are these genes related to the observable phenotype?

3. Why is there variability in the features of the phenotype, for example, the presence and severity of alpha thalassaemia is variable even for a given mutation and it seems likely that modifying genes are involved. Obvious candidates are genes encoding other protein components of heterochromatin.

4. What is the prevalence of ATR-X syndrome?
